# Management of Benzodiazepine Use in Youth and Young Adults: A Scoping Review

**DOI:** 10.1192/bjo.2024.212

**Published:** 2024-08-01

**Authors:** Nia Kakamousias, Laura Miller, Selene Etches, Melanie MacInnis

**Affiliations:** 1 Dalhousie University, Halifax, NS, Canada; 2 IWK Health, Halifax, NS, Canada

## Abstract

**Aims:**

Benzodiazepines are commonly used medications that have the potential for dependence and use disorder. Despite these harms, they are regularly prescribed and acquired from non-prescription sources. It has been established that benzodiazepine use is a widespread problem in youth and young adults. Little evidence exists to guide management of benzodiazepine use in this population. This scoping review aims to gather literature on the management of benzodiazepine use and identify the gaps in the literature to guide further research, particularly in youth and young adults.

**Methods:**

Methodology followed the Joanna Briggs Institute (JBI) and Preferred Reporting Items for Systematic Reviews and Meta-Analyses (PRISMA) extension for Scoping Reviews guidelines. MEDLINE (Ovid), Embase, Cochrane, and Cumulated Index to Nursing and Allied Health Literature (CINAHL) were searched, together with a search of the grey literature. A survey of experts in the field of addiction medicine was completed. Broad inclusion criteria were used to capture any available literature. Data were compiled using Covidence software, and two independent reviewers screened titles, abstracts, and full texts against the eligibility criteria. Data were extracted using a modified JBI data charting table. Descriptive statistics and a simple thematic analysis were performed to summarize the data collected.

**Results:**

Of the 835 papers retrieved, 104 papers published from December 1982 to March 2023 were included in the final review. Two of the papers included in this review pertained to youth and young adults. The rest of the papers were based on the adult population. Gradual dose reduction is the only method with evidence for efficacy in youth. Several therapies show efficacy in adults and could be future areas of research in youth, including benzodiazepine maintenance therapy, carbamazepine, gabapentin, pregabalin, trazodone, flumazenil slow infusion, and buprenorphine in various clinical contexts. Valproic acid, agomelatine, tricyclic antidepressants, paroxetine, buspirone, progesterone, cyamemazine, magnesium aspartate, clonidine, lithium, hydroxyzine, chlorpromazine, alpidem, captodiame, and ondansetron were deemed ineffective, unsafe in youth, or were not available for use in Canada. Topiramate, lamotrigine, oxcarbamazepine, phenobarbital, propranolol, baclofen, mirtazapine, and nicotinic acid had preliminary, low-quality evidence in adults, and would require further study.

**Conclusion:**

Benzodiazapine use disorder in youth is dangerous and common, and the lack of pharmacotherapeutic options has been deemed significant by our research team. The results of this review are promising in that they provide some further guidance on the management of this condition.

